# Personality traits and theory of mind: Performance data of a Spanish sample of university students

**DOI:** 10.1016/j.dib.2017.08.014

**Published:** 2017-08-30

**Authors:** José M. Gavilán, Juan Haro

**Affiliations:** Department of Psychology/CRAMC - Universitat Rovira i Virgili, Tarragona, Spain

## Abstract

This article allows consulting the performance data of 96 Spanish university students in two personality questionnaires and two theory of mind (ToM) tasks. Personality dimensions were evaluated through the OPERAS questionnaire (Vigil-Colet et al., 2013) [1], which evaluates global personality through 5 scales: *extraversion*, *agreeableness*, *conscientiousness*, *emotional stability*, *openness to experience*, and the ESQUIZO-Q questionnaire (Fonseca-Pedrero et al., 2010) [2], which assesses schizotypy by means of 10 subscales: *ideas of reference*, *magical thinking*, *unusual perceptual experiences*, *odd thinking and language*, *paranoid ideation*, *physical anhedonia*, *social anhedonia*, *odd behavior*, *lack of close friends*, and *excessive social anxiety*. The ability to attribute/infer mental states, i.e. ToM, was measured through two computerized tasks: the revised Reading the Mind in the Eyes (Baron-Cohen et al., 1997, 2001) [3], [4], and the Director's task (Keysar et al., 2000, 2003; Dumontheil et al., 2010) [5–7].

**Specifications Table**

**Table:** 

Subject area	*Psychology*
More specific subject area	*Personality traits, ToM abilities, individual differences*
Type of data	*Excel spreadsheet*
How data was acquired	*In the laboratory through individual computers. Participants answered computerized questionnaires and performed computerized ToM tasks.*
Data format	*Mean reaction times and number of errors from both ToM tasks, and factor scores on questionnaires’ scales.*
Experimental factors	*Director's task: Condition (Director or No-Director) and type of trial (Control or Experimental)*
Experimental features	*Director's task and Reading the mind in the eyes: mean response time and errors. Questionnaires: factors’ scores.*
Data source location	*The assessments were done in Tarragona, Spain.*
Data accessibility	*Data is with this article as Supplementary material*

**Value of the data**

• Our data may be useful for conducting individual statistical analyses and meta-analyses within the context of ToM and personality dimensions.
• Our data may serve to establish between-group differences with other Spanish age-cohorts or educational level samples, or with samples from other countries.
• Our data may be also useful to analyze gender differences in ToM and personality dimensions.
• Our data can also be compared with other cognitive or social performance data obtained in the same or different age-cohort (either Spanish or from other countries).

## 1 Data

The dataset is provided as an Excel file. Table 1 summarizes the data included in the file.

**Table 1:** Fields included in the data file.

Field name	Description
Participant	Participant's id
Age	Participant's age (in years)
Gender	Participant's gender (male, female)
DE_errors	Director's task: Number of errors in the Experimental trials of the Director condition. Range: 0–8.
DC_errors	Director's task: Number of errors in the Control trials of the Director condition. Range: 0–8.
DF_errors	Director's task: Number of errors in the Filler trials of the Director condition. Range: 0–32.
NDE_errors	Director's task: Number of errors in the Experimental trials of the No-Director condition. Range: 0–8.
NDC_errors	Director's task: Number of errors in the Control trials of the No-Director condition. Range: 0–8.
NDF_errors	Director's task: Number of errors in the Filler trials of the No-Director condition. Range: 0–32.
DE_rt	Director's task: Mean response time (in milliseconds) for the correct responses in the Experimental trials of the Director condition.
DC_rt	Director's task: Mean response time (in milliseconds) for the correct responses in the Control trials of the Director condition.
DF_rt	Director's task: Mean response time (in milliseconds) for the correct responses in the Filler trials of the Director condition.
NDE_rt	Director's task: Mean response time (in milliseconds) for the correct responses in the Experimental trials of the No-Director condition.
NDC_rt	Director's task: Mean response time (in milliseconds) for the correct responses in the Control trials of the No-Director condition.
NDF_rt	Director's task: Mean response time (in milliseconds) for the correct responses in the Filler trials of the No-Director condition.
EYES_errors	Reading the Mind in the Eyes: Number of errors. Range: 0–36
EYES_rt	Reading the Mind in the Eyes: Mean response time (in milliseconds) for the correct responses.
extraversion	Overall Personality Assessment Scale: score on Extraversion.
emotional_stability	Overall Personality Assessment Scale: score on Emotional Stability.
conscientiousness	Overall Personality Assessment Scale: score on Conscientiousness.
agreeableness	Overall Personality Assessment Scale: score on Agreeableness.
openness	Overall Personality Assessment Scale: score on Openness to Experience.
social_desirability	Overall Personality Assessment Scale: score on Social Desirability.
acquiescence	Overall Personality Assessment Scale: score on Acquiescence.
ideas_reference	Oviedo Schizotypy Assessment Questionnaire: score on Ideas of Reference. Range: 1–5.
magical_thinking	Oviedo Schizotypy Assessment Questionnaire: score on Magical Thinking. Range: 1–5.
unusual_perceptual_exp	Oviedo Schizotypy Assessment Questionnaire: score on Unusual Perceptual Experiences. Range: 1–5.
odd_thinking_lang	Oviedo Schizotypy Assessment Questionnaire: score on Odd Thinking and Language. Range: 1–5.
paranoid_ideation	Oviedo Schizotypy Assessment Questionnaire: score on Paranoid Ideation. Range: 1–5.
physical_anhedonia	Oviedo Schizotypy Assessment Questionnaire: score on Physical Anhedonia. Range: 1–5.
social_anhedonia	Oviedo Schizotypy Assessment Questionnaire: score on Social Anhedonia. Range: 1–5.
odd_behavior	Oviedo Schizotypy Assessment Questionnaire: score on Odd Behavior. Range: 1–5.
lack_close_friends	Oviedo Schizotypy Assessment Questionnaire: score on Lack of Close Friends. Range: 1–5.
social_anxiety	Oviedo Schizotypy Assessment Questionnaire: score on Excessive Social Anxiety. Range: 1–5.

## 2 Experimental design, materials and methods

### 2.1 Participants

The present data was obtained from 96 undergraduate students from the Universitat Rovira i Virgili (age range=17–33 years, mean age=20.75, SD=3.02; 70 females and 26 males). During the session, each participant completed an experimental task and filled in a set of questionnaires. The whole session lasted approximately 45 min. They received academic credits for their collaboration.

### 2.2 Experimental task

**Fig. 1:**
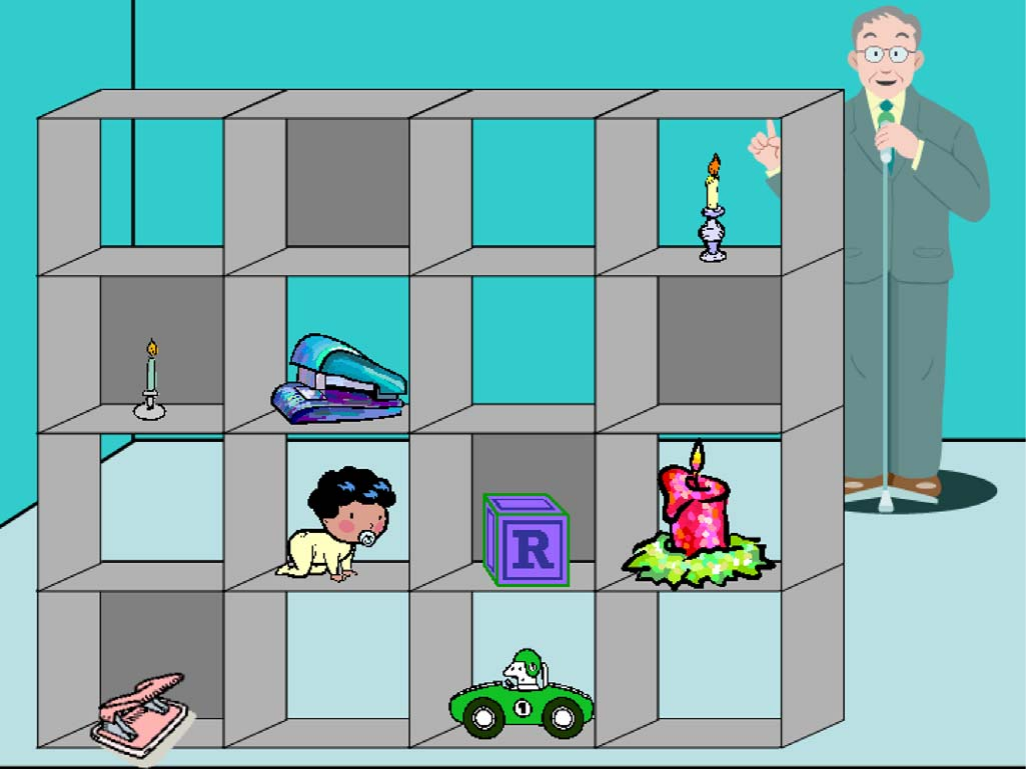
Director's task. Example of an Experimental trial from the Director condition. Participants heard the instruction “Move the small candle down”. The smallest candle (i.e., that located in the first slot of the second row) was occluded from the view of the director, so that the correct answer was to move down the smallest candle visible for the director (i.e., that located in the fourth slot of the first row).

**Fig. 2:**
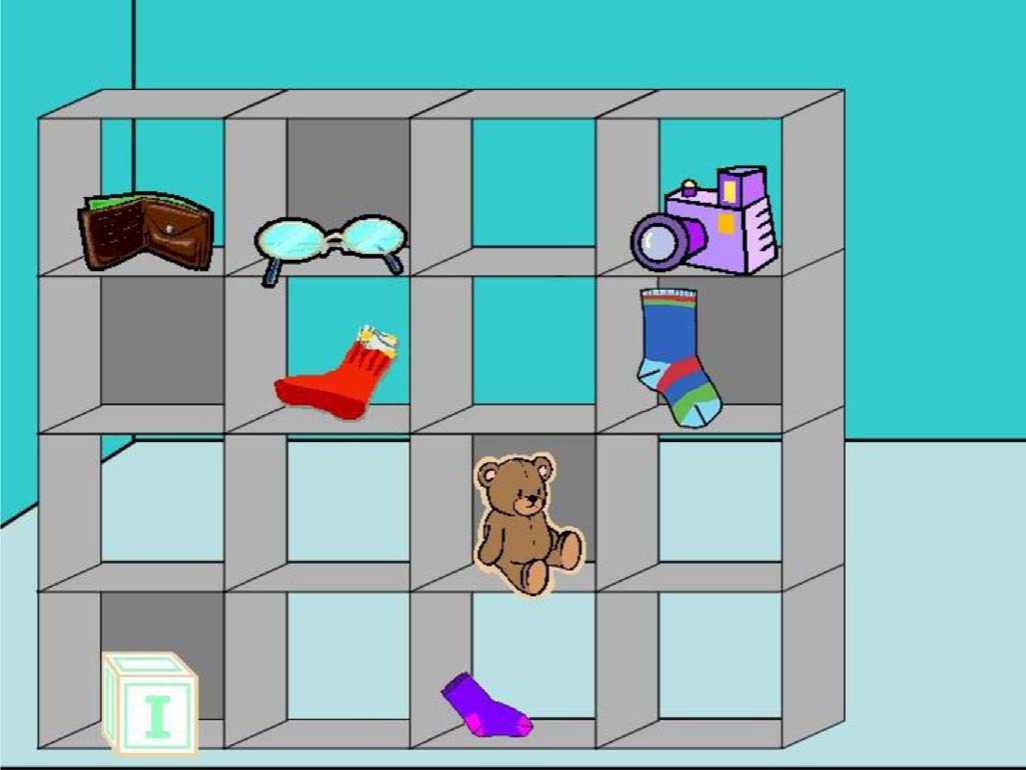
Director's task. Example of an Experimental trial from the No-Director condition. Participants heard the instruction “Move the large sock down”. The largest sock was placed in a slot with grey background (i.e., that located in the fourth slot of the second row), hence the correct answer was to move down the largest sock placed in a visible slot (i.e., that located in the second slot of the second row).

The experimental design involved two within-subject factors: condition (Director or No-Director) and type of trial (Control or Experimental). Participants completed a referential communication task, which was used in previous studies that investigated the development of theory of mind in late adolescents and adults [5], [6], [7]. The procedure was identical to that used in Dumontheil et al. [7]. The stimuli consisted of 32 pictures presented on a computer. Each picture showed a 4×4 set of shelves, with eight slots containing an object (e.g., a lipstick) and eight slots being empty. In each trial, participants were asked to move a given object of the shelves towards a specified direction (e.g., *move the lipstick down*). The instructions were presented through headphones. Participants were told that the instructions were given by the “director”, a character who stood on the other side of the shelves in the Director condition (and thus viewed the shelves from behind), but who was absent in the No-Director condition (see Fig. 1, Fig. 2). After the offset of the instruction, participants had to click with the mouse on the object they thought the director was referring to, and then to click on the appropriate slot on the shelves.

In the Director condition, participants were required to take into account the director's perspective. Importantly, in the trials of this condition, five slots of the shelves were occluded from the view of the director (i.e., those slots having a grey background). Thus, the correct response was to select the object that was visible for the director and was the best fit for the instruction. Experimental trials included a distractor object, which was the best fit for the instruction, but was not visible for the director. In Control trials, the arrangement of the objects was identical to that in the Experimental trials, but an irrelevant object replaced the distractor object. Finally, in filler trials, participants were asked to move objects that were placed only in visible slots. In the No-Director condition, the director was absent, and participants were asked to move only those objects placed in visible slots. Thus, instead of taking into account the director's perspective, participants had to ignore all objects in slots with a grey background. Experimental, control and filler trials were included in this condition. Participants completed the Director condition before the No-Director condition.

### 2.3 Questionnaires

The Reading the Mind in the Eyes is a social sensitivity (theory of mind) test developed by Baron-Cohen and colleagues [3], [4]. We implemented the revised version of the test [4] in the lab computers to run it as an online questionnaire. The test is made of 36 items, each of them showing a picture of a set of eyes depicting a thinking or feeling, and four words meaning emotions. The participant's task was to match, in a fixed-choice paradigm, the emotionality expressed by the eyes with the word that best described that thinking or feeling. During the completion of the test, participants could consult a booklet containing the definitions of the words that appeared in each item. The questionnaire was administered individually (see Section 3) and errors and response times were registered for each item.

The Oviedo Schizotypy Assessment Questionnaire [2] (ESQUIZO-Q) is a self-report questionnaire composed of 51 items in a 5-point Likert-type response format (1: Completely disagree; 5: Completely agree) designed to assess schizotypal traits in youth population. It is made of a total of 10 subscales, which are enumerated as follows (number of items, Cronbach's *α* obtained in this study, and an example item are provided for each subscale): Ideas of reference (four items, Cronbach's *α*=.70; e.g., *I think that I can detect hidden messages on TV or on the radio*)*,* Magical thinking (five items, Cronbach's *α*=.63; e.g., *My charms can make me pass an exam*)*,* Unusual perceptual experiences (seven items, Cronbach's *α*=.71; e.g., *I hear voices that others cannot hear*)*,* Odd thinking and language (six items, Cronbach's *α*=.83; e.g., *I find it difficult to concentrate on what I do*)*,* Paranoid ideation (five items, Cronbach's *α*=.75; e.g., *item 17: I think someone is plotting against me*)*,* Physical anhedonia (four items, Cronbach's *α*=.43; e.g., *When I'm showering I like to feel the water on my skin*)*,* Social anhedonia (five items, Cronbach's *α*=.53; e.g., *I like to help my friends and family when they need it*)*,* Odd behavior (four items, Cronbach's *α*=.76; e.g., *My friends say that the way I dress is strange*)*,* Lack of close friends (four items, Cronbach's *α*=.73; e.g., *I find it difficult to trust my relatives*) and Excessive social anxiety (seven items, Cronbach's *α*=.83; e.g., *I get nervous even when I'm with my friends*). These subscales are grouped into three general dimensions: *Reality distortion, Negative*, and *Interpersonal disorganization*. The Cronbach's *α* for the whole scale in this study was .88. The questionnaire was administered individually (see Section 3) and participants had to rate their agreement with each proposition (item).

The Overall Personality Assessment Scale (OPERAS) [1] is a brief self-reported personality questionnaire based on the big five personality factors. It is composed of 40 items that assess 5 uncorrelated traits. Such traits are represented in the following subscales (number of items, Cronbach's *α* obtained in this study, and an example item are provided for each subscale): Extraversion (seven items, Cronbach's *α*=.85; e.g., *I make friends easily*), Emotional stability (seven items, Cronbach's *α*=.82; e.g., *I feel comfortable with myself*), Conscientiousness (seven items, Cronbach's *α*=.76; e.g., *I am perfectionist*), Agreeableness (seven items, Cronbach's *α*=.69; e.g., *I respect others*), and Openness to experience (seven items, Cronbach's *α*=.68; e.g., *I like to visit museums*). Furthermore, the questionnaire includes a scale that controls for the main subject's response biases, i.e., social desirability and acquiescence. The Cronbach's *α* for the whole scale in this study was .78. The test was administered individually (see Section 3) in the psychology lab. Participants had to rate their agreement with each proposition (item) in a 1–5 Likert scale.

## 3 Procedure

Participants completed a total of four tasks, two of them assessing ToM capacities (Director's and Reading the Mind in the Eyes tasks), and two assessing personality dimensions (ESQUIZO-Q and OPERAS questionnaires). Prior to start any task, all participants signed an informed consent that specified the aim and details of the study. All tasks were implemented in computers sited in a quiet room in the psychology lab. Each participant did the four tasks individually always following the same sequence: (1) Director's task, (2) Reading the Mind in the Eyes task, (3) ESQUIZO-Q questionnaire and (4) OPERAS questionnaire. Overall, the experiment lasted about 45 min. An experimenter was always available in the control room to introduce and explain the instructions of each task.
