# Supplementary material for: Personality traits and theory of mind: Performance data of a Spanish sample of university students
Source: Data Brief. 2017 Aug 30;14:612–7. doi: 10.1016/j.dib.2017.08.014 (PMC5582375; doi:10.1016/j.dib.2017.08.014)
Supplement: Supplementary file 1 — Supplementary material [file mmc1.pdf]

Conflicts of Interest Statement

Manuscript title: PERSONALITY TRAITS AND THEORY OF MIND: PERFORMANCE DATA OF A SPANISH SAMPLE OF UNIVERSITY STUDENTS

The authors whose names are listed immediately below certify that they have NO affiliations with or involvement in any organization or entity with any financial interest (such as honoraria; educational grants; participation in speakers' bureaus; membership, employment, consultancies, stock ownership, or other equity interest; and expert testimony or patent-licensing arrangements), or non-financial interest (such as personal or professional relationships, affiliations, knowledge or beliefs) in the subject matter or materials discussed in this manuscript.

Author names:

JOSÉ M. GAVILAN
JUAN HARO

The authors whose names are listed immediately below report the following details of affiliation or involvement in an organization or entity with a financial or non-financial interest in the subject matter or materials discussed in this manuscript. Please specify the nature of the conflict on a separate sheet of paper if the space below is inadequate.

Author names:
